# Analysis of Circulating Waves in Tissue Rings derived from Human Induced Pluripotent Stem Cells

**DOI:** 10.1038/s41598-020-59803-9

**Published:** 2020-02-19

**Authors:** Lu Zhang, Junjun Li, Li Liu, Chao Tang

**Affiliations:** 1 Center for Quantitative Biology, Peking University, Beijing, 100871 China; 2 Peking-Tsinghua Center for Life Sciences, Peking University, Beijing, 100871 China; 3 School of Physics, Peking University, Beijing, 100871 China; 4 Department of Cardiovascular Surgery, Osaka University Graduate School of Medicine, 2-2 Yamadaoka, Suita, Osaka, 565-0871 Japan

**Keywords:** Biophysics, Systems biology

## Abstract

Developing more mature cardiomyocytes derived from human induced pluripotent stem cells is essential for cell transplantation and drug screening. In a previous study, we described a platform on which cardiomyocytes derived from human induced pluripotent stem cells (hiPSC-CMs) formed three-dimensional self-organized tissue rings. Within these rings, traveling waves of action potentials spontaneously originate and propagate for a long time. In order to understand the dynamic behavior of these waves, we developed a mathematical model for the circulation of the electrical signal in such rings. By using the restitution curves of the action potential and the conduction velocity we demonstrated the mechanisms underlying the steady circulation and the features dependent on velocity. The analytic result agreed well with the experimental data in the origination, propagation, and long-term behavior of traveling waves within self-organized tissue rings. The theoretical analysis of traveling waves may also provide a reference to the analysis of reentrant rhythms in hearts.

## Introduction

The discovery of induced pluripotent stem cells^1^ has made significant strides in the last ten years. However, cardiomyocytes derived from human induced pluripotent stem cells (hiPSC-CMs) display an immature, fetal like phenotype^2–4^. These characteristics could impede hiPSC-CMs’ applications in cell transplantation, drug screening, tissue engineering and regenerative medicine applications^3,5^. Mechanical and electrical stimulations are effective to achieve higher maturation of hiPSC-CMs. The addition of mechanical stimulation can increase contraction via hypertrophic pathways and the electrical pacing leads to enhanced cell–cell coupling and improved calcium handling^5^. However, these stimulation methods require complex supporting facilities. In addition, electrical stimulation could also change the pH value of the culture medium and generate toxic substances^5^.

**Figure 1:**
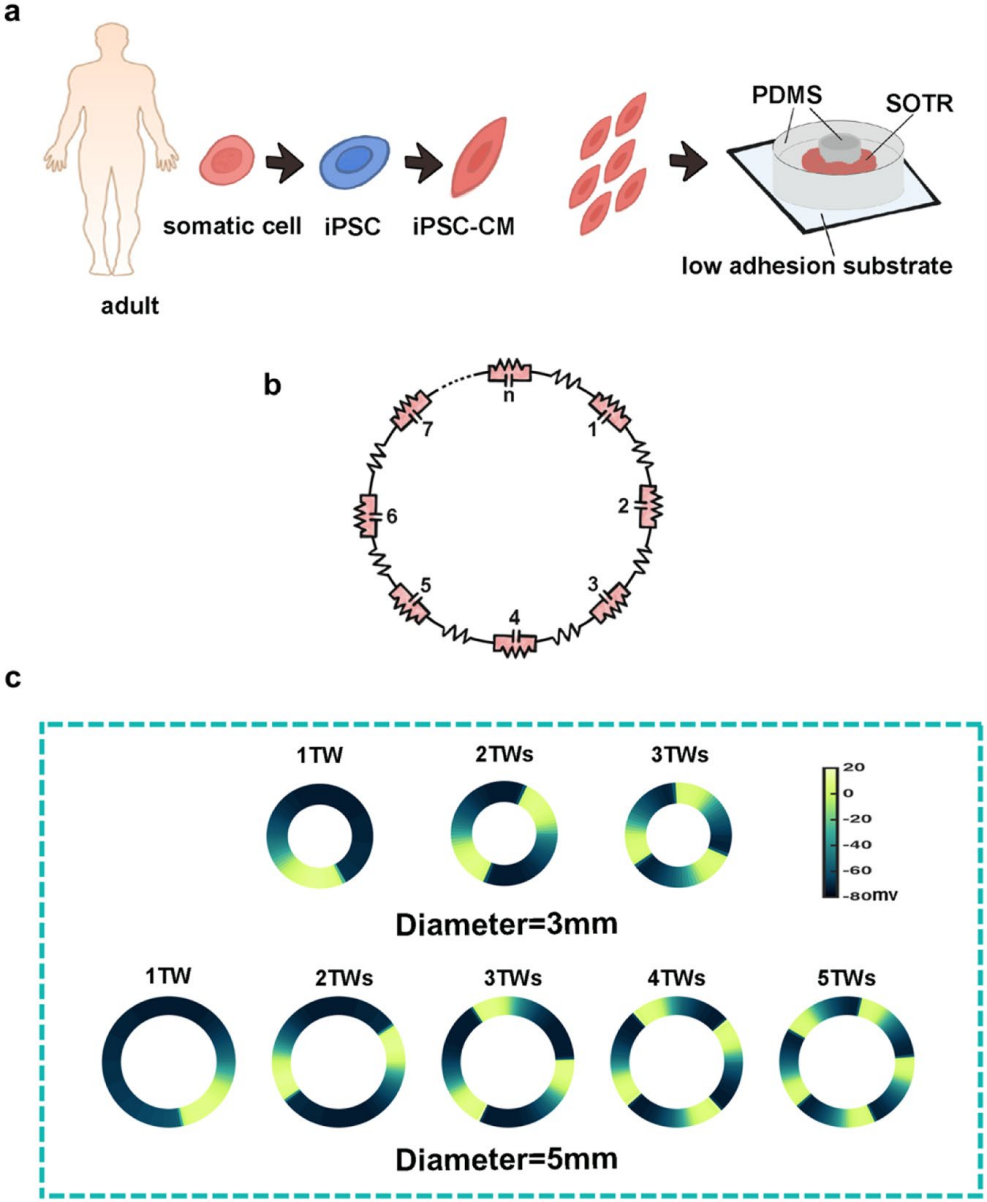
Generation of experimental cardiomyocytes rings derived from human induced pluripotent stem cells and the ring-shaped cable model in the simulation. (**a**) Schematic of hiPSC-CMs and self-organized tissue rings (SOTR) in the experiment (PDMS, polydimethylsiloxane). (**b**) Schematic of the ring-shaped cable model in the simulation. (**c**) Examples of different numbers of waves traveling around the 3- and 5-mm rings in simulations (TW, traveling wave).

In previous studies^6^, we created a platform on which hiPSC-CMs self-organized into three-dimensional tissue rings (SOTR), where action potentials spontaneously originated and propagated in the form of traveling waves (TWs) (Fig. 1a). Similar to the external electrical stimulation, traveling waves maintained electrical signals spreading unceasingly on the ring which continuously stimulated hiPSC-CMs and then made them more mature. While unlike the external electrical stimulation, owing to the ring’s special geometric structure, the natural boundary conditions made traveling waves spread continuously; it means that hiPSC-CMs were stimulated without external driving force. Our previous data showed that cardiac myofilaments, trained by traveling waves, were arranged densely along the ring orientation, with the length of sarcomeres becoming significantly longer and the cardiac specific proteins, such like troponin T and α-actinin, being up-regulated. These experimental results indicated that TWs in SOTRs can promote maturation of hiPSC-CMs^6^.

In addition to the above experimental phenomena, we also found interesting dynamic behavior of traveling waves. It was noted that these waves were similar to the reentrant excitation phenomenon in the heart. Over one hundred years ago, George Mines demonstrated that a ring of cardiac muscle from a dogfish could sustain a continually circulating wave of contraction^7^. In 1988 Lawrence Frame and Michael Simson also reported a reentrant excitation in a ring of canine heart muscle, and found complex fluctuations in the cycle times^8^. Similarly, many researches also use mammalian heart cells to construct cell rings and then produce electrical signal waves by applying external stimulation. Because of the heterogeneity of mammalian heart cells, it is difficult to observe steadily propagating waves for the long term. However, in our systems the heterogeneity of hiPSC-CMs was low and we could observe long-term behavior of traveling waves. Moreover, we found that there were multiple traveling waves propagating on the ring and unstable waves could be caused by the long-term culture.

Therefore, we constructed a one-dimensional cable model to elucidate the propagation and long-term behavior of traveling waves. There are quite a number of researches on wave propagation in hearts both in experiments^9–12^ and theories^13–19^. Especially, Leon Glass and his colleagues described specific recovery and restitution properties during the wave propagation and identified three essential functions: the conduction velocity restitution curve, the phase resetting curve, and the action potential restitution curve^20^.

In this study, by utilizing simulation methods, we found constraints of stable wave transmission in our experimental systems. By using restitution properties mentioned above, we calculated the maximum number of traveling waves in rings with different diameters which conformed to simulation results. Furthermore, we also found the reason why longer culture time in experiments led to unstable waves. Finally, we indicated the crucial factor which determined the speed of traveling waves under different experimental conditions. To conclude, we established a mathematical model to explain the dynamic mechanism of various traveling waves reported in our previous studies. These theoretical results may also provide a reference to the analysis of reentrant rhythms in hearts.

## Materials and Methods

### Culture of hiPSC-CMs and generation of self-organized tissue rings

Human iPSCs were cultured and differentiated at 37 °C according to previously published methods^2,21^. Briefly, after differentiation, cardiomyocyte colonies were collected and single cells were isolated using protease solution^2^. The dissociated cardiomyocytes were filtered and resuspended in serum-supplemented cardiac differentiation medium and then plated in PDMS (SYLGARD 184) culture wells with 8 mm inner diameters and pillars with various diameters in the center. The plated cardiomyocytes aggregated and formed a thick tissue ring within 2 days (Fig. 1a). As indicated by the genetically-encoded calcium indicator GCaMP3, we found activation and looped propagation on the ring by using a fluorescence microscope equipped with a CCD camera as described previously^6^. GCaMP3 was excited at the wavelength from 450 nm to 490 nm, and the images were recorded with 8 × 8 binning of CCD pixels at 30 frames/s. The raw data were exported to ImageJ and MATLAB for further analysis.

### Mathematical model

We constructed a discontinuous one-dimensional ring model (Fig. 1b), in which each individual cell was based on the classic Priebe & Beuckelmann membrane model^22^ with an added hyperpolarization-activated current^23^ (I_f_) and a reduced inward rectifier K^+^ current (I_K1_), because of the differences between human ventricular cells and the hiPSC-CMs^3^. The membrane model included the following ionic currents: I_Na_ (sodium current), I_Ca_ (calcium current), I_to_ (transient outward current), I_K_ (delayed rectifier K^+^ current), I_K1_ (inward rectifier K^+^ current), background currents (I_Ca,b_ and I_Na,b_), I_NaK_ (Na^+^-K^+^ pump), I_NaCa_ (Na^+^/Ca^+^ exchanger), and I_f_ (funny current). We set the parameters as follows (in nS/pF): g_Na_ = 8, g_Ca_ = 0.064, g_to_ = 0.35, g_K_ = 0.1, g_K1_ = 1.17, g_Na,b_ = 0.001, g_Ca,b_ = 0.00085, g_NaK_ = 1.3, g_NaCa_ = 1000, and g_f_ = 0.08. To increase the computational efficiency and stability, we also applied Panfilov’s reformulated model based on the Priebe & Beuckelmann model^24^. It is important to note that, unlike normal human ventricular cells, hiPSC-CMs are able to beat spontaneously, so they are similar to sinoatrial node cells. Therefore, the hyperpolarization-activated current (I_f_) had to be added, and the inward rectifier K^+^ current (I_K1_) had to be reduced. There is strong evidence that a reduced I_K1_^25,26^ and a prominent I_f_^23^ contribute to the automaticity of CMs. Neighboring cells were connected by a pure resistance representing gap junctions and we used the cable equation (Eq. 1) to describe the conduction of electrical signals^27^.

1 $$\begin{array}{c}\frac{\partial {\rm{V}}}{\partial {\rm{t}}}=\frac{1}{{{\rm{C}}}_{{\rm{m}}}}\frac{{\rm{a}}}{2{{\rm{R}}}_{{\rm{i}}}{{\rm{R}}}_{{\rm{CG}}}}\frac{{\partial }^{2}{\rm{V}}}{\partial {{\rm{x}}}^{2}}-\frac{1}{{{\rm{C}}}_{{\rm{m}}}}{{\rm{I}}}_{{\rm{total}}}\\ {{\rm{R}}}_{{\rm{i}}}={{\rm{R}}}_{{\rm{myo}}}+\frac{{{\rm{R}}}_{{\rm{g}}}}{\Delta x}\end{array}$$

where V is the membrane potential, C_m_ is the membrane capacitance, I_total_ is the sum of individual ionic currents in a single cell, a is the cell radius, Δx is the length of a single cell, R_CG_ is the ratio between capacitive and geometric areas, R_myo_ is the myoplasmic resistance, and R_g_ is the gap-junction resistance. Changes in the longitudinal axial resistivity were introduced by varying R_g_ between 0.3 and 1.5 Ω. Gap-junction resistances were homogeneously modulated everywhere in the ring. The entire cable was composed of 30–600 cells of realistic dimensions (100 μm long, 11 μm in diameter) with an R_myo_ of 0.001 Ω/μm. Finally, the periodic boundary condition V(1) = V(N + 1) was imposed on a one-dimensional cable, both ends of which were connected, creating a ring-like structure to simulate the self-organized tissue rings in the experiments. The initial membrane potential around a ring was V(1, 2 … N) = V_random_ (all cells randomly set in different phases of the action potential).

## Results

### Maximum numbers of traveling waves in rings with different diameters

**Figure 2:**
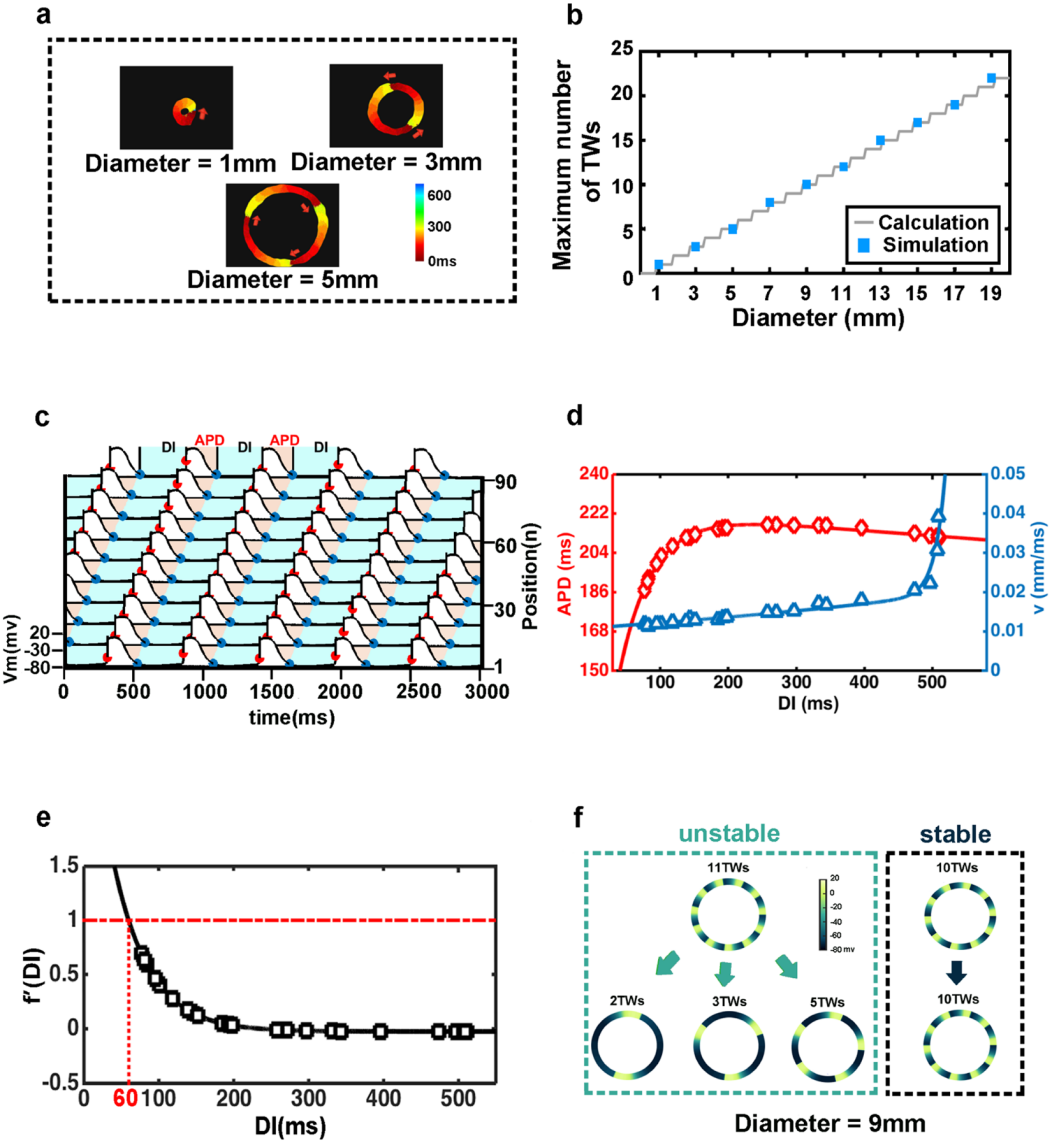
Maximum number of traveling waves, the definition of the action potential restitution function and the conduction velocity restitution function. (**a**) Activation map of GCaMP3-positive in SOTRs with 1TW in 1mm-ring, 2TWs in 3mm-ring, and 3TWs in 5mm-ring on day 6 (experimental data). The red arrows indicate the propagation direction of the traveling waves. (**b**) Maximum number of traveling waves in simulation (blue squares) and calculation (grey line). (**c**) Definition of action potential duration (APD) and diastolic interval (DI). (**d**) Curves of the action potential restitution function (red diamonds: simulation result; red line: fitting result) and the conduction velocity restitution function (blue triangles: simulation result; blue line: fitting result). (**e**) The derivative of the action potential restitution function (black line: calculation result; black squares: the corresponding derivative of the simulation result) and the threshold of the stable condition (red line). (**f**) Examples of stable and unstable traveling waves in a 9-mm diameter ring (simulation).

Using the one-dimensional ring-shaped computer model and random initial conditions, we obtained different numbers of waves in rings of various diameters (Fig. 1c). The total number of cells in each ring in the model was proportional to the diameter of the rings in the experiments (Fig. 2a). According to simulations (fixed gap-junction resistance) and experiments^6^ (day 6 in culture), we found that the maximum number of waves at different ring diameters increased linearly (Fig. 2b). In order to explain this phenomenon, two crucial functions were introduced into the model: the action potential restitution function and the conduction velocity restitution function.

There is a consensus that the recovery properties for the conduction velocity (v) and the action potential duration (APD) are determinants of the dynamics of wave propagation in cardiac tissue^28^. In the mathematical model, we defined the start of the APD as the point of maximum rate of change of the membrane potential, and the end at the point when 90% of the minimum membrane potential was reached^29^. The time interval from the end of the preceding action potential to the beginning of the present action potential was defined as the diastolic interval (DI) (Fig. 2c). Conduction speeds were calculated using the propagation time of the wave front over the perimeter of one ring. In the simulations, starting with random initial conditions we maintained the cell-cell coupling strength (corresponding to a fixed time of culture in the experiments) and changed the total number of cells in the ring (corresponding to different ring diameters in the experiments), and then collected data on APD, DI, and v after the system reached stable states. Integrating all the data from all simulation conditions, we obtained the action potential restitution function APD = f(DI) and the conduction velocity restitution function v = c(DI) (Fig. 2d).

Considering one wave circulating around a ring, we obtained following relationship^17^:

2 $${\rm{DI}}({\rm{x}})={\int }_{{\rm{x}}-{\rm{L}}}^{{\rm{x}}}\frac{{\rm{ds}}}{{\rm{v}}({\rm{s}})}-{\rm{APD}}({\rm{x}}-{\rm{L}}).$$

In Eq. 2 L represents the perimeter of the ring. And, using the action potential and the conduction velocity restitution functions, Eq. 2 can be rewritten as Eq. 3^17^.

3 $${\rm{DI}}({\rm{x}})={\int }_{{\rm{x}}-{\rm{L}}}^{{\rm{x}}}\frac{{\rm{ds}}}{{\rm{c}}({\rm{DI}}({\rm{s}}))}-{\rm{f}}({\rm{DI}}({\rm{x}}-{\rm{L}})).$$

Equation 3 is an integral-delay equation. And the derivative of APD = f(DI) < 1 is the condition for the steady-state solution (Fig. 2e).

Considering the real situation in which waves of different numbers could appear in a fixed-diameter ring, we used “n” to represent the number of waves, and then obtained the relationship between the conduction speed and the conduction time in the ring.

4 $${\rm{\pi }}d={\rm{v}}\times ({\rm{APD}}+{\rm{DI}})\times {\rm{n}}.$$

In Eq. 4, d represents the diameter of the ring. Rearranging Eq. 4, it was evident that when the value of d was fixed, the maximum number of waves only depended on the minimum DI.

5 $${\rm{n}}=\frac{{\rm{\pi }}}{{\rm{c}}({\rm{DI}})\times ({\rm{f}}({\rm{DI}})+{\rm{DI}})}{\rm{d}}.$$

On the other hand, the steady-state condition required the derivative of the function APD = f(DI) < 1, so from $\frac{{\rm{dAPD}}}{{\rm{dDI}}}={\rm{f}}{\prime} ({\rm{DI}})$ function we obtained the minimum DI, the value of which was 60 ms with a fixed value of gap-junction resistivity (Fig. 2e). According to Eq. 5, when the minimum DI was fixed, the relationship between the diameter (d) and the maximum number of waves (n_max_) in a ring of this diameter was linear, and the slope of this linear function was 1.1487. In the experiments, we were only able to use rings of three different diameters due to instrumental restrictions^6^. However, in the numerical simulations, we were able to consider larger diameters and obtain stable maximum numbers of waves at diameters from 1 to 19 mm. The simulation results of maximum numbers of waves agreed well with the calculated results (Fig. 2b). For instance, in the simulations, when diameter of the ring was set to 9 mm and the total cell number to 280, the stable maximum number of waves was 10 in the calculation. In simulations, when we set 11 waves in the initial condition, they did not maintain circulation and randomly broke down into fewer waves, while if we set 10 waves initially, circulation was maintained for a long time (Fig. 2f).

**Figure 3:**
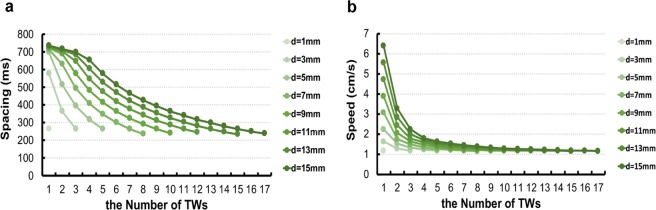
Spacings and speeds of traveling waves computed based on the restitution functions. (**a**) The spacings of traveling waves of different numbers in varied diameters calculated from Eq. 5 and restitution functions. (**b**) The speeds of traveling waves in different diameters and numbers computed from restitution functions.

In addition, the Eq. 5 demonstrated the relationship among the number of traveling waves (n), the diameter of the rings (d) and the diastolic interval time of cells (DI) (fixed gap-junction resistance). According to the Eq. 5, when the variables d and n are fixed, the value of DI can be calculated analytically. Furthermore, using the action potential restitution function and the conduction velocity restitution function, the spacing and the speed of the traveling waves with fixed d and n can also be calculated. It means that based on the Eq. 5 and the restitution functions we can compute spacing and speeds of expected numbers of waves in the rings (Fig. 3a,b).

### Analytical stability criteria for the degree of cellular coupling

**Figure 4:**
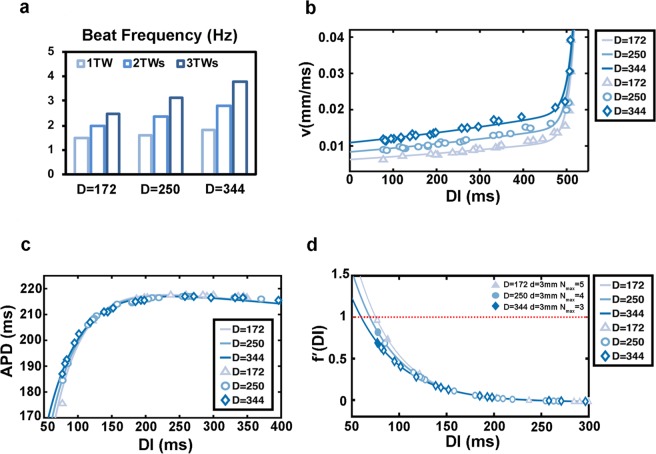
Increased strength of gap junctions leads to instability of traveling waves. (**a**) Beating frequency of cells in different numbers of traveling waves at three strengths of cell-cell coupling (d = 3 mm) (simulation). (**b**–**d**) The conduction velocity restitution curves (**b**), action potential restitution curves (**c**), and derivative of the action potential restitution function (**d**) with different gap-junction strengths. Triangle, circular and rhombus: simulation results; lines: fitting results.

In the experiments, we observed that the number of waves decreased and the beating frequency increased with the increase of culture time^6^. We then investigated the potential causes for these phenomena. From other previous studies and ours, with the longer culture time, the strength of gap junctions between adjacent cells increased^27,30,31^. To simulate this effect in our model, we set three different strengths of gap junctions and in order to simplify this parameter we used the diffusion coefficient D $({\rm{D}}=\frac{{\rm{a}}}{\,2{{\rm{R}}}_{{\rm{i}}}{{\rm{R}}}_{{\rm{CG}}}},\,{\rm{units}}:{\rm{S}}\cdot {{\mu }m}^{2})$ to represent it. The values of D were 172, 250, and 344, from weak to strong. The simulation results showed that when D was increased the beat frequency of the waves gradually increased (Fig. 4a). Consistently, stronger gap junctions resulted in faster speeds of all traveling waves, irrespective of wave numbers and ring diameters (Fig. 4b). We plotted the action potential restitution functions under the weak, medium, and strong gap-junction conditions (Fig. 4c), and found that there was a slight change in the curvature in the region of small DI with increasing junction strength. This trend caused the derivative curve of the action potential restitution function to bend downwards. This trend can cause the waves of large wave number to become unstable (Fig. 4d). For instance, if we fixed the ring diameter to 3 mm, the maximum number of waves was 5 for the weak and 4 for the middle strength junctions, while that number was 3 for strong junctions (Fig. 4d). Thus, the change in derivative curves explained the experimental results that waves with large wave number became unstable and the wave number gradually decreased with increased culture time.

### The key factor affecting the speed of traveling waves

**Figure 5:**
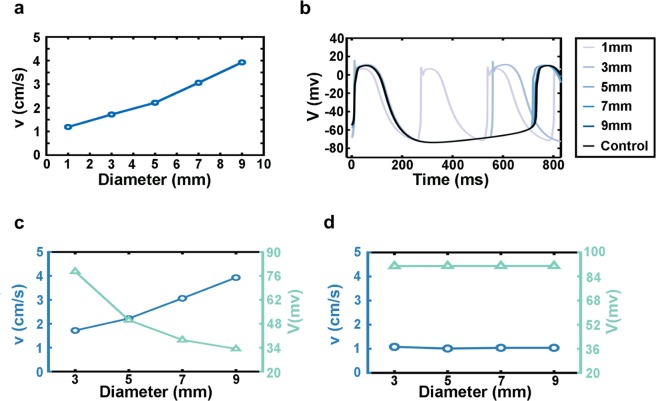
The relationship between the traveling wave speed and the membrane potential difference. (**a**) The relationship between the speed of 1 wave and the ring diameter in simulations. (**b**) Membrane potential curves of single-cell in rings maintaining 1 wave with different diameters. (**c**) Relationships among wave speeds, potential difference between neighboring cells just before activation, and ring diameters (blue: wave speed; green: potential difference). (**d**) Ring diameters does not affect the speed of 1 traveling wave when cells lose the hyperpolarization-activated current (blue: speed; green: potential difference). (**a**–**d**: simulation).

Both in experiments and simulations, the speed of waves varied under different conditions such as the number of waves and the diameter of rings. First, we maintained the gap junction strength and the perimeter of rings unchanged. When we set the diameter to 3 mm, 1-, 2-, and 3-wave cases occurred. As the number of waves increased, their speeds decreased – 1 wave propagated faster than 2 or 3 waves – and this trend was consistent in experiments^6^ and simulations. Then, focusing on 1 wave in different diameter rings, we found that the propagation speed in the rings increased with the diameter both in simulations (Fig. 5a) and experiments^6^.

An important factor which determined speeds of traveling waves was electric potential difference of adjacent cells at the wave front. All hiPSC-CMs beat spontaneously because of the existence of the hyperpolarization-activated current (I_f_), which gradually elevated cells’ membrane potential. In the absence of waves, hyperpolarization-activated current helped cells start depolarization and this duration was longer. However, in the case with waves, because spontaneous beating interval of cells was larger than the interval time resulted from neighbor cells’ activation, the beginning of cells’ depolarization was mainly caused by their neighbors. Since the time they activated by their neighbors after the onset time of hyperpolarization, the hyperpolarization also played a role to reduce the potential difference between two adjacent cells. For one traveling wave in different ring diameters, a larger diameter led cells closer to their own depolarization threshold (Fig. 5b), resulting in a smaller potential difference and thus a faster wave propagation (this is also the reason why 1 wave was faster than 2 waves in the same diameter ring: the duration of hyperpolarization was shorter with 2 waves than 1 wave, so the potential difference between cells was larger with 2 than with 1 wave). In addition, with a 1-mm pillar, the cells took the shortest time to hyperpolarize and their maximum depolarizing membrane potential was lower than with the larger pillars. It has long been known that a decrease in sodium channel protein expression or the sodium channel that does not fully recover from preceding depolarization-repolarization process can lead to a decrease in the corresponding ionic current, which reduces the value of the maximum depolarizing membrane potential and thus reduces the propagation speed of the electrical signal^27^. Therefore, the speed of 1 wave in the 1 mm ring was the slowest (Fig. 5a,b) (and the slowest speed of 3 waves in the 3 mm ring can be explained by the same mechanism).

**Figure 6:**
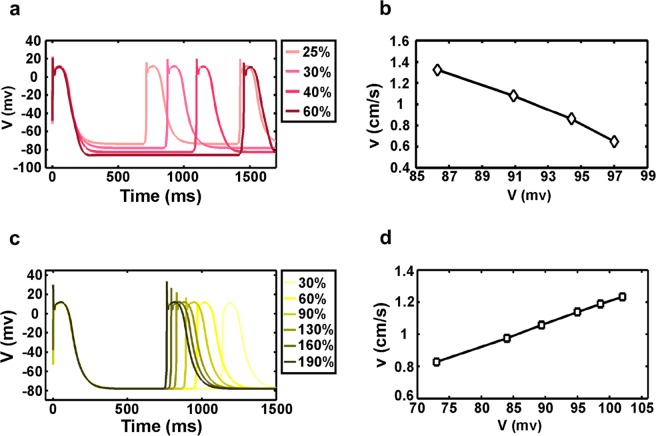
Membrane potential difference directly affects traveling wave speed under no hyperpolarization-activated current conditions. (**a**) Single cell membrane potential curves of different I_K1_. (**b**) Increased potential difference caused by I_K1_ leads to lower wave speed. (**c**) Single-cell membrane potential curves under different I_Na_ conditions. (**d**) Increased potential differences caused by I_Na_ leads to higher wave speed. (**a**–**d**: simulation).

To test the above hypothesis, we performed three further tests. First, we deleted the hyperpolarization-activated current (I_f_) in all cells in rings with diameter >3 mm, which controlled the potential differences between yet-to-be-activated cells and their neighbors activating them (Fig. 5c). Without this current, the electric potential difference between the activating cell and its neighboring to-be-activated cell at the wave front would be the same for rings of different diameters. Indeed, we found that in this case the speed of one wave was constant under different ring diameters (Fig. 5d). Second, we turned the cells’ excitability (with no hyperpolarization-activated current) with the proportion of inward rectifier K^+^ currents (I_K1_) (Fig. 6a). The resting state potential was affected by the concentration of potassium ions and the gradient of potential difference was established by adjusting the current of potassium ions. We measured speeds of one wave in 3 mm diameter ring, and found decreased speeds corresponding to increased potential differences (Fig. 6b). This result agreed well with our conclusion that a difference in membrane potential was the factor that direct affected the speed of waves in rings with different diameters. Third, we increased or decreased the sodium channel current of the cells (Fig. 6c) while maintaining their excitability. The previous research has demonstrated that the added sodium channel current could increase velocity of electrical signal propagation^27^. And in our test, we reached the same conclusion. In addition, because of the changed sodium channel current, the maximum membrane potential varied when the cell depolarized and resulted in change in the potential difference between the yet-to-be-activated cell and the neighbors that activated it. We found that, with an increase of the potential difference, the propagation speed of the waves also increased (Fig. 6d). It should be noted that although both the sodium current and the inward rectifier K^+^ current led to changes in the potential difference between cells, these two changes had different effects on the speed of waves. The decreased maximum membrane potential caused a decrease in the potential difference, leading to a slower speed, while the increased minimum membrane potential caused a decrease in the potential difference, leading to a faster speed. In conclusion, these three tests demonstrated that different wave speeds in rings of diverse diameters or various numbers of waves were caused by differences in the electrical potential between activated and activating cells.

## Summary

Excitable chemical media in annular geometries can easily maintain circulating waves once they are initiated^32,33^. However, because of heterogeneities it is difficult to produce persistent circulating waves in a ring of cardiac cells. Although in many idealized settings the conservation of circulating waves is expected, in more realistic situations there is no priori reason to expect conservation of the numbers of circulating waves. Especially in experimental and clinical systems in cardiac electrophysiology, circulating waves often start or stop in a paroxysmal manner. In previous studies, we demonstrated a variety of traveling waves which arose spontaneously and maintained for a long time in self-organized tissue rings. And these spontaneously generated waves dramatically promoted the maturity of hiPSC derived cardiomyocytes, enhanced cardiac gene expression, and improved the Ca^2+^-handling properties^6^. In this work, based on the computational simulation and the theoretical analysis, we demonstrate that the speed of electrical signal propagation depend on the difference in electrical potential and the time available for a cell to recover after the preceding excitation. We find that circulating traveling waves can be sustained or lost depending on the derivative of the action potential restitution function. Moreover, the strength of gap junctions can change the curvature of the action potential restitution function, thereby determining the stability of traveling waves.

**Figure 7:**
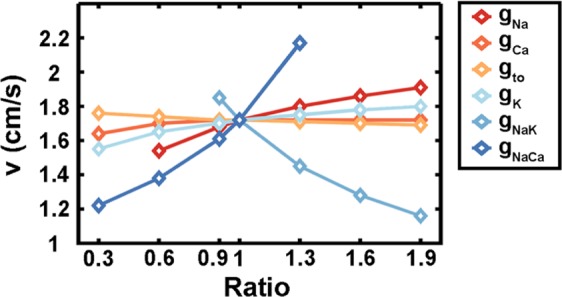
Different ionic current strengths lead to different speeds of one traveling wave in the 3-mm diameter ring. The standard ratio is 1 and the parameters are described in the mathematical model section. If the ratio is less than 1, the corresponding ionic current decreases, while if the ratio is greater than 1, the corresponding ionic current increases (simulation).

In conclusion, we have found that a large number of distinct traveling waves can be found in annular geometry both in experiments and simulations. Traveling waves promote the frequency-dependent structural and functional maturation of hiPSC-CMs, offering a supplementary approach to traditional maturation methods which depend on electrical or mechanical stimulation. Furthermore, because of the close relationship between the speed of a traveling wave and the strength of each ionic channel current^34^ (Fig. 7), drug response on relatively mature cardiomyocytes in the ring could be more precise and stable. The variation of wave speed can be used to predict the ionic channels affected by a tested drug. Moreover, the theoretical analysis of traveling waves presented here also provides a reference for the analysis of reentrant rhythms in the heart.

## Supplementary information

Supplementary Information.

Supplementary Information 2.

Supplementary Information 3.

Supplementary Information 4.
