# Supplementary material for: Analysis of Circulating Waves in Tissue Rings derived from Human Induced Pluripotent Stem Cells
Source: Sci Rep. 2020 Feb 19;10:2984. doi: 10.1038/s41598-020-59803-9 (PMC7031392; doi:10.1038/s41598-020-59803-9)
Supplement: Supplementary file 4 — Supplementary Information 4. [file 41598_2020_59803_MOESM4_ESM.pdf]

Supplementary Information

Analysis of Circulating Waves in Tissue Rings derived from Human Induced Pluripotent Stem Cells

Model Equations

The model we used refers to classic Priebe & Beuckelmann membrane model[1] and modified PB model by Bernus et al[2] and Hyperpolarization-activated current model by Verkerk et al[3].

Equilibrium Potentials:

$$E_{Na} = \frac{RT}{F}\ln\left(\frac{[Na^+]_e}{[Na^+]_i}\right) \quad (S\text{-}1)$$

$$E_{Ca} = \frac{RT}{2F}\ln\left(\frac{[Ca^{2+}]_e}{[Ca^{2+}]_i}\right) \quad (S\text{-}2)$$

$$E_{to} = \frac{RT}{F}\ln\left(\frac{0.043\times[Na^+]_e+[K^+]_e}{0.043\times[Na^+]_i+[K^+]_i}\right) \quad (S\text{-}3)$$

$$E_K = \frac{RT}{F}\ln\left(\frac{[K^+]_e}{[K^+]_i}\right) \quad (S\text{-}4)$$

Na^+ current:

$$I_{Na} = g_{Na} \times m^3 \times v^2 \times (V_m - E_{Na}) \quad (S\text{-}5)$$

$$\alpha_m = \frac{[0.32\times(V_m+47.13)]}{1-\exp^{[-0.1\times(V_m+47.13)]}} \quad (S\text{-}6)$$

$$\beta_m = 0.08 \times \exp^{\frac{-V_m}{11}} \quad (S\text{-}7)$$

$$\frac{dm}{dt} = (1-m)\times\alpha_m - m\times\beta_m \quad (S\text{-}8)$$

$$v_\infty = 0.5 \times [1 - \tanh(7.74 + 0.12 \times V_m)] \quad (S\text{-}9)$$

$$\tau_v = 0.25 + 2.24 \times \frac{1-\tanh(7.74+0.12\times V_m)}{1-\tanh[0.07\times(V_m+92.4)]} \quad (S\text{-}10)$$

$$\frac{dv}{dt} = \frac{v_\infty(V_m)-v}{\tau_v(V_m)} \quad (S\text{-}11)$$

Ca^{2+} current:

$$I_{Ca} = g_{Ca} \times d_\infty \times f \times f_{Ca} \times (V_m - E_{Ca}) \quad (S\text{-}12)$$

$$d_\infty = \frac{\alpha_d}{\alpha_d+\beta_d} \quad (S\text{-}13)$$

$$\alpha_d = \frac{14.98\times\exp^{\{-0.5\times[\frac{(V_m-22.36)}{16.68}]^2\}}}{16.68\times\sqrt{2\pi}} \tag{S-14}$$

$$\beta_d = \frac{0.1471-5.3\times\exp^{\{-0.5\times[\frac{(V_m-6.2)}{14.93}]^2\}}}{14.93\times\sqrt{2\pi}} \tag{S-15}$$

$$\alpha_f = \frac{0.00687}{1+\exp^{-\frac{(6.1546-V_m)}{6.12}}} \tag{S-16}$$

$$\beta_f = \frac{0.069\times\exp^{[-0.11\times(V_m+9.825)]}+0.011}{1+\exp^{[-0.278\times(V_m+9.825)]}} + 0.000575 \tag{S-17}$$

$$\frac{df}{dt} = (1-f)\times\alpha_f - f\times\beta_f \tag{S-18}$$

$$f_{Ca} = \frac{1}{1+\frac{[Ca^{2+}]_i}{0.0006}} \tag{S-19}$$

Transient outward current:

$$I_{to} = g_{to}\times r_\infty\times t_o\times(V_m - E_{to}) \tag{S-20}$$

$$r_\infty = \frac{\alpha_r}{\alpha_r+\beta_r} \tag{S-21}$$

$$\alpha_r = \frac{0.5266\times\exp^{[-0.0166\times(V_m-42.2912)]}}{1+\exp^{[-0.0943\times(V_m-42.2912)]}} \tag{S-22}$$

$$\beta_r = \frac{0.00005186\times V_m+0.5149\times\exp^{[-0.1344\times(V_m-5.0027)]}}{1+\exp^{[-0.1348\times(V_m-0.00005186)]}} \tag{S-23}$$

$$\alpha_{to} = \frac{0.00005612\times V_m+0.0721\times\exp^{[-0.173\times(V_m+34.2531)]}}{1+\exp^{[-0.1732\times(V_m+34.2531)]}} \tag{S-24}$$

$$\beta_{to} = \frac{0.0001215\times V_m+0.0767\times\exp^{[-1.66\times10^{-9}\times(V_m+34.0235)]}}{1+\exp^{[-0.1604\times(V_m+34.0235)]}} \tag{S-25}$$

$$\frac{dt_o}{dt} = (1-t_o)\times\alpha_{t_o} - t_o\times\beta_{t_o} \tag{S-26}$$

$$\tau_{t_o}(V_m) = \frac{1}{P\times\alpha_{t_o}(V_m)+P\times\beta_{t_o}(V_m)} \tag{S-27}$$

$$t_{o\infty}(V_m) = \frac{\alpha_{t_o}\times(V_m-V_{shift})}{\alpha_{t_o}(V_m-V_{shift})+\beta_{t_o}(V_m-V_{shift})} \tag{S-28}$$

$$\frac{dt_o}{dt} = \frac{t_{o\infty}-t_o}{\tau_{t_o}} \tag{S-29}$$

Delayed rectifier K^+ current:

$$I_K = g_K\times X^2\times(V_m - E_K) \tag{S-30}$$

$$X_{\infty} = \frac{0.972}{1+\exp^{-(2.036+0.0834\times V_m)}} \tag{S-31}$$

$$\tau_X = 380 \times \exp^{-\frac{(V_m+25.5)^2}{156}} + 166 \times [1 + \tanh(0.558 + 0.0169 \times V_m)] \tag{S-32}$$

$$\frac{dX}{dt} = \frac{X_{\infty}-X}{\tau_X} \tag{S-33}$$

Inward rectifier K^+ current:

$$I_{K_1} = g_{K_1} \times K_{1_{\infty}} \times (V_m - E_K) \tag{S-34}$$

$$K_{1_{\infty}} = \frac{\alpha_{K_1}}{\alpha_{K_1}+\beta_{K_1}} \tag{S-35}$$

$$\alpha_{K_1} = \frac{0.1}{1+\exp^{[0.06\times(V_m-E_K-200)]}} \tag{S-36}$$

$$\beta_{K_1} = \frac{3\times\exp^{0.0002\times(V_m-E_K+100)}+\exp^{0.1\times(V_m-E_K-10)}}{1+\exp^{-0.5\times(V_m-E_K)}} \tag{S-37}$$

Background current:

$$I_{Ca,b} = g_{Ca,b} \times (V_m - E_{Ca}) \tag{S-38}$$

$$I_{Na,b} = g_{Na,b} \times (V_m - E_{Na}) \tag{S-39}$$

Na^+-K^+ pump:

$$I_{NaK} = g_{NaK} \times f_{NaK} \times f'_{NaK} \tag{S-40}$$

$$f_{NaK} = \frac{1}{1+0.1245\times\exp^{-0.0037\times V_m}+0.0365\times\sigma\times\exp^{-0.037\times V_m}} \tag{S-41}$$

$$f'_{NaK} = \frac{1}{1+(\frac{10}{[Na^+]_i})^{1.5}} \times \frac{[K^+]_e}{[K^+]_e+1.5} \tag{S-42}$$

$$\sigma = 0.1428 \times (\exp^{\frac{[Na^+]_e}{67.3}} - 1) \tag{S-43}$$

Na^+/Ca^{2+} exchanger:

$$I_{NaCa} = g_{NaCa} \times f_{NaCa} \tag{S-44}$$

$$f_{NaCa} = \frac{[Na^+]_i^3\times[Ca^{2+}]_e\times\exp^{0.013\times V_m}-[Na^+]_e^3\times[Ca^{2+}]_i\times\exp^{-0.024\times V_m}}{(669921+[Na^+]_e^3)\times(1.38+[Ca^{2+}]_e)\times(1+0.1\times\exp^{-0.024\times V_m})} \tag{S-45}$$

Hyperpolarization-activated current:

$$I_f = y \times g_f \times (V_m - E_f) \tag{S-46}$$

$$\frac{dy}{dt} = \frac{y_\infty - y}{\tau_y} \qquad (S\text{-}47)$$

$$\tau_y = \frac{1000}{0.36 \times \frac{(V_m+148.8)}{\exp^{0.066\times(V_m+148.8)}-1} + 0.1 \times \frac{(V_m+87.3)}{1-\exp^{-0.21\times(V_m+87.3)}}} - 54 \qquad (S\text{-}48)$$

if $V_m < -80\text{mV}$:

$$y_\infty = \frac{0.99921}{1+\exp^{\frac{V_m+97.134}{8.1752}}} + 0.01329 \qquad (S\text{-}49)$$

if $V_m \geq -80\text{mV}$:

$$y_\infty = \exp^{-\frac{V_m}{12.861}} \times 0.0002501 \qquad (S\text{-}50)$$

Concentration of major ions in and out of the cell membrane (units: mM)

$[Ca^{2+}]_i$	$[Ca^{2+}]_e$	$[Na^+]_i$	$[Na^+]_e$	$[K^+]_i$	$[K^+]_e$
0.0004	2	10	138	140	4

Other parameters:

$R = 8.314\text{J/K}$;$T = 273 + 37(\text{K})$;$F = 96.5/\text{mmol}$;$P = \frac{1}{1.7}$;$V_{shift} = -4\text{mV}$;$E_f = -22\text{mV}$

References

1. Priebe, L. & Beuckelmann, D. J. Simulation study of cellular electric properties in heart failure. *Circ Res* **82**, 1206–1223 (1998).
2. Bernus, O., Wilders, R., Zemlin, C. W., Verschelde, H. & Panfilov, A. V. A computationally efficient electrophysiological model of human ventricular cells. *Am. J. Physiol. - Hear. Circ. Physiol.* (2002). doi:10.1152/ajpheart.00731.2001
3. Verkerk, A. O., Van Borren, M. M. G. J. & Wilders, R. Calcium transient and sodium-calcium exchange current in human versus rabbit sinoatrial node pacemaker cells. *Sci. World J.* **2013**, (2013).
